# Pneumocephalus and pneumorachis after blunt chest trauma without spinal fractures: a case report

**DOI:** 10.1186/s13256-019-2208-3

**Published:** 2019-10-25

**Authors:** Etienne Allard, Jean Selim, Benoit Veber

**Affiliations:** Department of Anesthesiology and Critical Care, Rouen University Hospital, Rouen, France

**Keywords:** Pneumocephalus, Pneumorachis, Blunt chest trauma, Case report

## Abstract

**Background:**

Pneumocephalus and pneumorachis, presence of air inside the skull and spinal canal, are mostly seen after neurosurgical procedures and neuraxial anesthesia. They have also been described after penetrating trauma, but never after blunt trauma without adjacent bone fractures.

**Case description:**

We present the case of an 85-year-old white male patient admitted to our intensive care unit after a high velocity car accident. On site clinical evaluation showed normal consciousness with 15/15 Glasgow Coma Scale after a short initial loss of consciousness. The patient was first sent to a nearby hospital where a whole-body computed tomography scan revealed pneumocephalus and pneumorachis and an important left hemopneumothorax with pneumomediastinum with extensive subcutaneous emphysema. The state of the patient quickly worsened with hemorrhagic shock. The patient was sent to our intensive care unit; upon neurosurgical evaluation, no surgical indication was retained due to the absence of skull and spine fracture.

A computed tomography scan performed on day 6 showed total regression of the pneumocephalus and pneumorachis. A follow-up computed tomography scan performed on day 30 revealed no intracranial bleeding or stroke, but a left pleural hernia between ribs 5 and 6. Due to respiratory complications, our patient could not be weaned from ventilator support for a proper neurological examination.

Our patient’s state finally worsened with septic shock due to ventilator-acquired pneumonia leading to multiple organ failure and our patient died on day 37.

**Conclusions:**

This is the first case report to describe pneumorachis and pneumocephalus following blunt trauma with pneumothorax, but no spinal or skull fractures. The mechanism that is probably involved here is a migration of air with subcutaneous emphysema and a pleural hernia into the spinal canal. However, in cases of pneumorachis or pneumocephalus, skull fractures need to be investigated as these require surgery and appropriate vaccination to prevent meningitis.

## Introduction

Pneumorachis is defined as the presence of air inside the spinal canal. It is mostly an iatrogenic complication of spine surgery or neuraxial anesthesia [1]. It is also a rare complication of thoracic trauma. Most reported cases showed pneumorachis happened after penetrating thoracic trauma or they were associated with spinal fractures [2, 3].

We report the case of a patient who presented with pneumorachis and pneumocephalus after a blunt thoracic trauma without spinal fractures or skull fractures. It is the only such case described so far.

## Case description

An 85-year-old white male patient was initially taken care of by an emergency medical team for a trauma following a car accident. The car exited the road and underwent five somersaults. There were no other victims involved. On primary evaluation, the patient presented a head trauma with loss of consciousness, a left ear lobe trauma, and a left thoracic trauma with emphysema. Consciousness restored spontaneously with a Glasgow Coma Score of 15/15 after a few minutes.

**Fig. 1:**
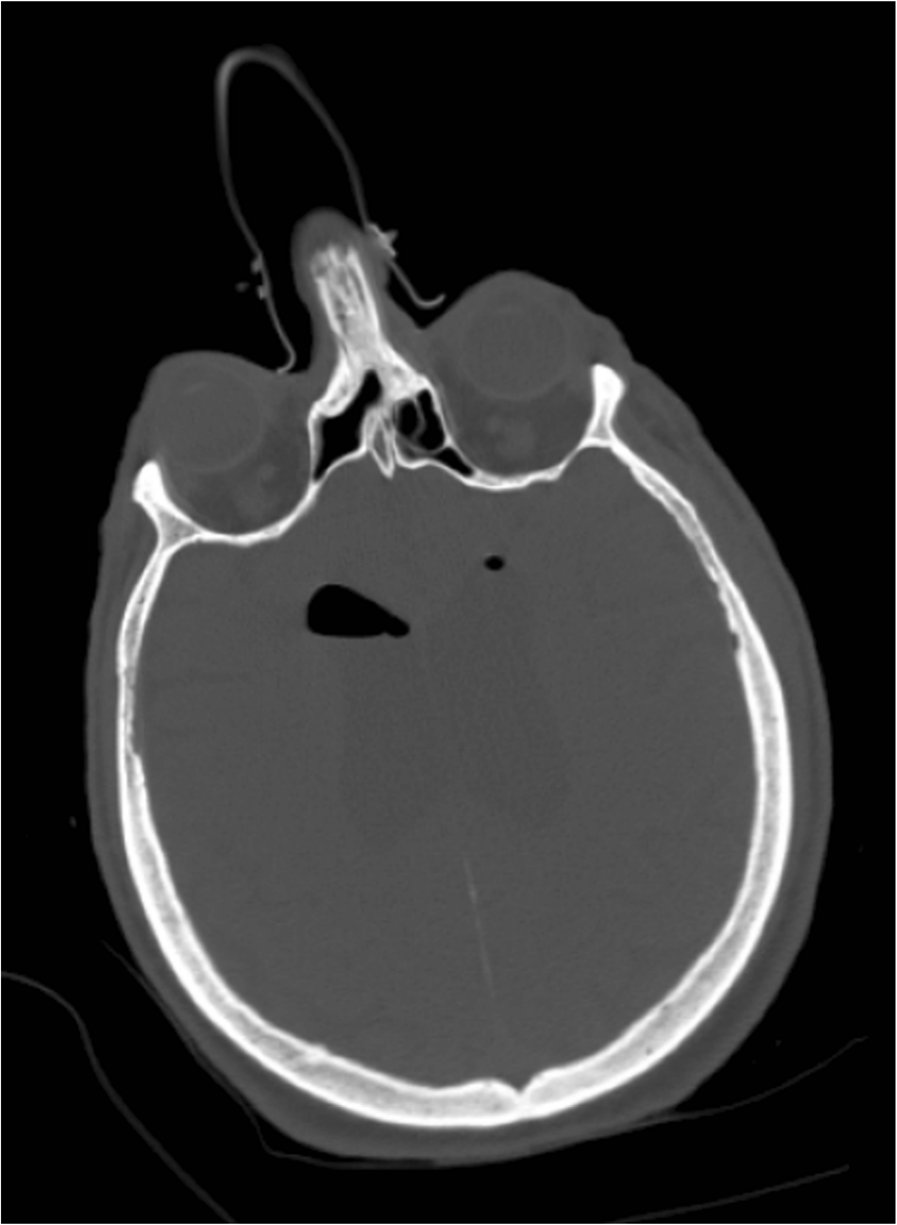
Initial brain computed tomography scan, axial view, showing pneumocephalus inside the lateral ventriculi. There are no associated fractures of the skull or the sinus walls

**Fig. 2:**
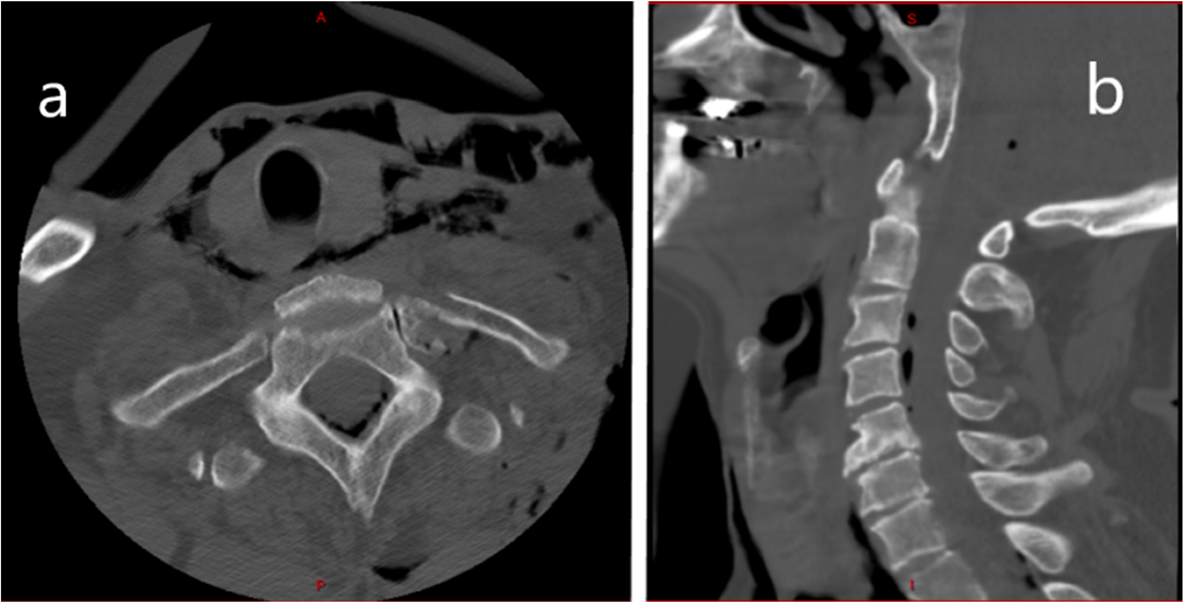
Initial cervical spine computed tomography, axial (**a**) and sagittal (**b**) view, showing pneumorachis on multiple levels

The patient was sent to the nearest hospital where a whole-body computed tomography (CT) scan was performed. A brain CT scan revealed air inside both lateral ventriculi and in the basal cisterns (Fig. 1). There were no associated skull fractures or intracranial bleeding. There was also perimedullar air in the spine at levels C3–C4 and C7–T1 (Fig. 2). No spinal fractures were seen. There were posterior fractures of all left ribs, anterior fractures of the fourth and fifth left ribs, and a left non-compressive hemopneumothorax. The left scapula was fractured. Soft tissue emphysema ranged from the left thorax to the neck with retropharyngeal extension. There was a pneumomediastinum. A CT scan showed no sign of tracheal or bronchial fistula.

After the CT scan, the patient’s hemodynamic and respiratory state worsened. The patient was intubated; norepinephrine infusion and blood transfusion were initiated. A left thoracic drainage was performed and drew 500 mL of blood. The patient was then transferred to our surgical intensive care unit (ICU) for thoracic surgery due to the displacement of the left costal fractures.

On arrival in our ICU, our patient presented with hemorrhagic shock due to parietal thoracic bleeding and hemothorax. Our patient was on high doses of norepinephrine. Transfusion was continued with red blood cells (RBC), fresh frozen plasma (FFP), and fibrinogen. Costal osteosynthesis of left ribs 3–9 was performed by thoracotomy and a new thoracic drainage was put in place, which allowed the control of the shock.

After reviewing the brain CT scan and examination by the neurosurgical team, there were no signs of skull fracture to account for the pneumocephalus thus no neurosurgical indication was retained.

A control CT scan was performed on day 6 and showed total regression of the pneumocephalus and the pneumorachis. The evolution was marked by multiple ventilator-acquired pulmonary infections requiring the pursuit of invasive ventilation and sedation. Multiple attempts of sedation withdrawal were performed but full neurological recovery could never be obtained due to agitation or a rapid need for sedation for ventilatory purposes. A follow-up CT scan performed on day 30 revealed no intracranial bleeding or stroke, but a left pleural hernia between ribs 5 and 6.

Our patient’s state finally worsened with septic shock due to ventilator-associated pneumonia leading to multiple organ failure with acute respiratory distress syndrome requiring prone positioning, acute kidney failure on continuous hemodialysis, and major hemodynamic failure despite high doses of norepinephrine. Our patient died on day 37.

## Discussion

### Mechanisms of pneumorachis and pneumocephalus

Pneumorachis has often been described in association with pneumothorax or pneumomediastinum. In a post-traumatic setting, it is mostly reported after penetrating trauma or associated with spinal fractures [2, 3]. The most frequent initial mechanism is an increase in intra-alveolar pressure (acute asthma, recurrent vomiting, or closed thoracic trauma). Barotrauma and alveolar rupture allow air migration along the bronchial tree up to the mediastinum. The collected air then separates the mediastinal pleura from the aorta and the parietal pleura from the spine; therefore, it enters the epidural space via the intervertebral foramina. The suddenness, more than the size of the pneumothorax, induces this initial increase in intra-alveolar pressure [4].

Pneumocephalus is mostly caused directly by opening of the skull, be it after traumatic skull fractures or iatrogenic after surgery. In this traumatic setting, skull base fractures need to be searched because of a communication with the upper airways and consequently a high risk of infections. Pneumocephalus can also be caused by the migration of air from the spinal perimedullar or subarachnoid spaces. And, finally, one case report showed pneumocephalus associated with pneumothorax without pneumorachis. The involved mechanism is the migration of air along the carotid sheath into the cranial cavity [5]. In the present case, we hypothesize that pneumocephalus is the consequence of air migration after pneumorachis following closed thoracic trauma.

The present case reports none of the main causes of pneumocephalus and pneumorachis. It is interesting to know that those conditions can be present without basal skull or spinal fractures needing surgical procedures.

### Neurological complications

Most studies do not report any neurological complication associated with pneumorachis. It can cause local pain or headaches [1]. In all reported post-traumatic cases, pneumorachis reabsorbed spontaneously. Only one study reports monoplegia of a limb with hypoesthesia. Even in this case, pneumorachis reabsorbed and the neurological deficit resolved spontaneously before surgery for spinal fractures [4]. One case of compressive postoperative pneumorachis causing cauda equina syndrome was reported. The patient fully recovered after being reoperated for air removal [6].

In the present case, our patient presented initial loss of consciousness which recovered spontaneously, probably due to a brain concussion. Radiological findings showed total reabsorption of spinal and intracranial air at day 6. Unfortunately, total recovery of consciousness could not be achieved during our patient’s stay because of the need for prolonged sedation for ventilatory purposes. Thus persistent neurological complications could not be assessed.

## Conclusion

The present case showed a pneumorachis and pneumocephalus after a closed thoracic trauma. It is the first case where no associated spinal or skull base fractures have been described. We therefore assume that the physiopathological mechanism involves an extension of the pneumothorax. Even though it reabsorbed spontaneously with apparently no sequels, this entity is interesting for the intensivist and surgeon taking care of traumatized patients. In fact, post-traumatic pneumocephalus requires systematic examination for skull base fractures that might need surgical procedures. In the absence of skull base fractures, pneumocephalus can be accounted for by thoracic trauma. This means that no other procedures may be indicated. Association with pneumorachis might be an additional hint toward this mechanism but does not rule out the need for an initial thorough skull base examination by the radiologist.

## Data Availability

Not applicable.
